# Extreme rainfall events and cooling of sea turtle clutches: Implications in the face of climate warming

**DOI:** 10.1002/ece3.7076

**Published:** 2020-12-17

**Authors:** Jacques‐Olivier Laloë, Jamie N. Tedeschi, David T. Booth, Ian Bell, Andy Dunstan, Richard D. Reina, Graeme C. Hays

**Affiliations:** ^1^ School of Life and Environmental Sciences Deakin University Geelong Vic. Australia; ^2^ School of Biological Sciences The University of Queensland Brisbane Qld Australia; ^3^ Queensland Department of Environment and Science Townsville Qld Australia; ^4^ Queensland Department of Environment and Science Queensland Parks and Wildlife Service and Partnerships Brisbane Qld Australia; ^5^ School of Biological Sciences Monash University Clayton Vic. Australia

**Keywords:** climate change, green sea turtle, hatching success, incubation temperature, marine turtles, precipitation, sex ratio

## Abstract

Understanding how climate change impacts species and ecosystems is integral to conservation. When studying impacts of climate change, warming temperatures are a research focus, with much less attention given to extreme weather events and their impacts. Here, we show how localized, extreme rainfall events can have a major impact on a species that is endangered in many parts of its range. We report incubation temperatures from the world's largest green sea turtle rookery, during a breeding season when two extreme rainfall events occurred. Rainfall caused nest temperatures to drop suddenly and the maximum drop in temperature for each rain‐induced cooling averaged 3.6°C (*n* = 79 nests, min = 1.0°C, max = 7.4°C). Since green sea turtles have temperature‐dependent sex determination, with low incubation temperatures producing males, such major rainfall events may have a masculinization effect on primary sex ratios. Therefore, in some cases, extreme rainfall events may provide a “get‐out‐of‐jail‐free card” to avoid complete feminization of turtle populations as climate warming continues.

## Graphical abstract

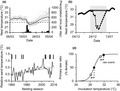

## 1 INTRODUCTION

Sea turtles are an iconic group with temperature‐dependent sex determination (TSD), where the sex of an individual is determined by the temperature experienced during the thermosensitive period (TSP) while eggs are incubating (Miller, 1997). For all sea turtle species, males are produced at low incubation temperatures and females at high incubation temperatures. A serious threat to sea turtles is that climate warming is raising incubation temperatures of nests and so causing increasing feminization of hatchling sex ratios (Glen & Mrosovsky, 2004; Hays et al., 2003; Jensen et al., 2018) as well as increasing hatchling mortality (Laloë et al., 2017; Monsinjon et al., 2019; Pike, 2014). Unless there is some other mechanism to reduce incubation temperatures, ultimately complete feminization of populations will lead to their extinction.

Yet as well as warming, it is also expected that the frequency of storms and associated extreme rainfall events will vary as part of climate change (CSIRO & BOM, 2018; Easterling et al., 2000; Kerr, 2011; Schiermeier, 2011; Trenberth, 2011). Extreme weather events were shown to have significant impacts on the physiology and ecology of plants (Gutschick & BassiriRad, 2003) as well as a range of animal taxa (Santidrián Tomillo et al., 2020; Ujvari et al., 2016). Potentially, therefore, different aspects of climate change, that is, warming versus increased extreme rainfall events, may have contrasting impacts on sea turtle incubation conditions.

While it is known that rainfall in general acts to cool the sand at incubation depths (Houghton et al., 2007; Lolavar & Wyneken, 2015) and has an impact on primary sex ratios (Lolavar & Wyneken, 2017, 2020), the impact of extreme rainfall or extreme drought has received little attention (but see Santidrián Tomillo et al., 2020; Ujvari et al., 2016). Here, we report nest temperatures at the world's largest green turtle (*Chelonia mydas*) rookery at Raine Island, Australia, during a breeding season that experienced two extreme rainfall events, that is, periods of time during which >500 mm of rain fell over five consecutive days. Recently, the extreme female‐biased sex ratio skew at this rookery was highlighted (Jensen et al., 2018), with >99% of juvenile turtles being females. Therefore, at this globally important rookery, climate warming and entire feminization of the hatchling production is a grave threat.

## 2 METHODS

### 2.1 Study site

Raine Island (11°35′S, 144°02′E) lies 80 km off the coast of Queensland and is located on the outer edge of the Great Barrier Reef. It is a small coral cay (approximately 1,800 m in circumference) that is vegetated with grasses and low scrubs but has no trees that could shade parts of the nesting beach. The sea turtle breeding season typically extends from November to March (Fuentes et al., 2010), during which the estimated mean number of females nesting varies widely between 5,000 and 70,000 depending on the particular season (Mast et al., 2011). The climate is typical wet/dry tropical with the warmer wet period occurring during December to March, and the cooler dry period occurring in June‐August (Limpus et al., 2003).

### 2.2 Nest temperatures

As part of a long‐term monitoring program, we deployed 124 temperature loggers (model DS1921H‐F5, Maxim Integrated, San Jose, California, USA, precision = 0.0625°C, accuracy = 0.5°C) into nests over the course of three excursions to Raine Island during the 2018/2019 nesting season. We deployed data loggers on October 19, 2018 (*n* = 45), December 1, 2018 (*n* = 52) and 12/13 February 2019 (*n* = 27). We placed loggers in the middle of clutches during oviposition and recorded temperatures hourly. Loggers were left in nests during the whole incubation process and were retrieved after hatchling emergence.

Prior to the experiment, we checked the accuracy of all temperature loggers in a water bath set at hourly intervals of 20, 25, 30, 35, and 40°C. This confirmed that loggers were accurate to 0.5°C, as specified by the manufacturer.

### 2.3 Environmental data

We downloaded rainfall data from the website of the Australian Government Bureau of Meteorology (http://www.bom.gov.au). Rainfall data are available as observations of daily rainfall. Measurements are nominally made at 9 a.m., local time, and record the total rainfall for the previous 24 hr. Since no weather stations are deployed on Raine Island, we used data from the nearest weather station, which is situated at Lockhart River Airport (approximately 150 km southeast of Raine Island).

To assess how rainfall‐linked drops in temperatures affected nest temperatures, we removed steep declines and subsequent increases in daily mean nest temperatures from our time‐series and filled resulting gaps using a linear interpolation. We propose that the observed difference in nest temperature between a nest with a sudden drop in nest temperature and a nest with the drop removed is due to the extreme rainfall event.

To examine how sand temperatures have changed at our study site in recent decades, we digitized sand temperature reconstructions during the nesting season for Raine Island from Jensen et al. (2018).

### 2.4 Sex ratio estimations

For several reasons, it may not be straightforward to reliably estimate hatchlings sex ratios from sand temperatures. First, the time during incubation when sex is determined is still not well defined. Pioneering experiments in the 1980s showed that the TSP for sex determination occurs somewhere in the middle third of embryonic development (Yntema & Mrosovsky, 1982). More recently, evidence has started to emerge that the TSP might only be a few days during the middle third of development (Porter, 2020). Second, for many populations, the relationship between temperature and sex ratio is not well defined, and so often generic relationships are used (Hays et al., 2017; Laloë et al., 2014). Hence rather than trying to estimate nest sex ratios, we instead simply report if rain could shift nest temperatures from more female‐producing to more male‐producing temperatures.

### 2.5 Nesting seasonality

Green sea turtle nesting season on Raine Island typically extends from November to March, with a peak in December/January (Fuentes et al., 2010). We modeled the nesting season using a normal distribution centered on December/January and approximated the percentage of nests that were affected by each extreme rainfall using this distribution. We also estimated how many extra males were produced as a result of the extreme rainfall event that occurred in March 2019 using a mean number of eggs per clutch = 104.3 (*SD* = 24.85; Limpus, 2008) and a mean emergence success = 60.6% (*SD* = 3.4; Dunstan & Robertson, 2017).

### 2.6 Statistical analyses

We performed the statistical analyses in R version 3.6.1 (R: A language and environment for statistical computing. R Foundation for Statistical Computing, Vienna, Austria, https://www.R‐project.org).

## 3 RESULTS

### 3.1 Cooling effect of rainfall

**FIGURE 1:**
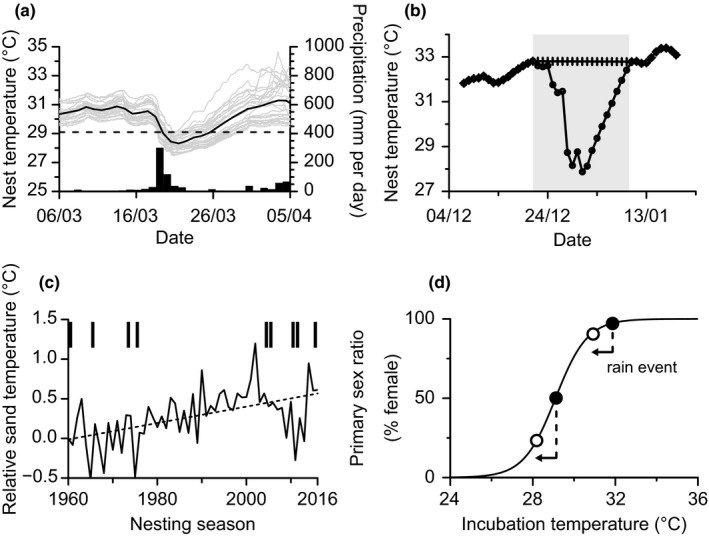
(a) Drops in nest temperatures (gray lines) coincided with extreme rainfall events (black bars). For example, 519 mm of rain fell between 19 and 23 March 2019 and nest temperatures dropped by an average 2.2°C (*n* = 27 nests). The black line represents daily mean temperatures for all nests. (b) Rainfall‐linked drops in sand temperature affected nest temperatures. Mean nest temperature during the twenty days that followed an extreme rainfall event (light gray area) was 30.7°C (●), whereas it would have been 32.4°C in the absence of rainfall (+). In the most extreme case, the mean temperature during the twenty days that followed an extreme rainfall event was lowered by 3.1°C due to a rainfall‐linked cooling event (mean drop = 1.1°C, *n* = 79 nests). (c) Sand temperatures (given relative to 1960; solid black line) increased on Raine Island between 1960 and the present. The dashed line is the regression line (*R* ^2^ = 0.26, *F_1,55_* = 19.22, *p* < .01). Vertical bars indicate nesting seasons during which >500 mm of rain fell over five consecutive days. (d) A 1.1°C decrease in mean nest temperature (horizontal arrows) during the TSP likely causes more male hatchlings to be produced in a nest. The generic relationship between nest temperature and sex ratio is from Hays et al., 2017

During our study, two extreme rainfall events occurred. Plotting nest temperatures alongside rainfall shows that sudden drops in nest temperatures coincided with extreme rainfall events recorded at the nearest weather station (Figure 1a). The first extreme rainfall event occurred at the end of December 2018, and the maximum drop in temperature for each nest averaged 4.3°C (*n* = 52 instrumented nests, min = 1.3°C, max = 7.4°C). The second extreme rainfall event occurred in March 2019, and the maximum drop in nest temperature averaged 2.3°C (*n* = 27 instrumented nests, min = 1.0°C, max = 2.9°C; Figure 1a,b). Of significance, in many nests incubation temperatures dropped below 29.0°C, which is the typical pivotal temperature for TSD (i.e., the temperature at which a 50:50 sex ratio is expected; Hays et al., 2017). Therefore, the sex ratios of nests that were incubating during this time likely changed due to the extreme rainfall event.

### 3.2 Implications in the face of climate change

Long‐term reconstructions of sand temperature on Raine Island suggest warming since 1960 (Figure 1c). In addition, empirical measurements show that rainfall events of similar magnitude to those recorded in our study have occurred approximately every 6 years at our field site (Figure 1c). However, as part of climate change, changes in rainfall patterns and intensity are projected for Australia. Australia's Commonwealth Scientific and Industrial Research Organisation (CSIRO) and Bureau of Meteorology (BOM) predict increases in intense heavy rainfall throughout Australia, particularly for short duration extreme rainfall events (CSIRO & BOM, 2018). While it is difficult to project exactly how rain patterns will change in the future, we can already assess the impact different scenarios will have on primary sex ratios (Figure 1d).

## 4 DISCUSSION

Understanding how variable environmental conditions such as the weather impact species with TSD continues to be a prolific field of research (Fuentes et al., 2011; Godfrey et al., 1996; Hays et al., 2003; Lolavar & Wyneken, 2015; Mrosovsky et al., 1984; Schoeman et al., 2014; Shine & Elphick, 2001). For sea turtles, previous studies have focused on the cooling effect seasonal precipitation patterns have on reproductive success and primary sex ratios (e.g., Laloë and Esteban et al., 2015; Lolavar & Wyneken, 2015). For examples, it was shown that more leatherback turtle (*Dermochelys coriacea*) males are produced during a wet year on Grenada, in the Caribbean, than during a dry year (Houghton et al., 2007). Impacts of rainfall are also observable within a same year, and more male turtles are produced during the colder and wetter months of the nesting season than during the warmer and drier months (Mrosovsky et al., 1984).

In contrast to seasonal—and relatively predictable—precipitation, the impact of extreme weather on primary sex ratios has received little attention (Santidrián Tomillo et al., 2020).

Here, we assumed that precipitation recorded at the nearest weather station is representative of precipitation at our field site, given the synchronicity of temperature drops in our study nests with extreme rainfall events recorded at the nearest weather station. We have shown that heavy rainfall can cool sea turtle nests appreciably and so likely increases the proportion of male hatchlings. For example, if mean incubation temperatures during the middle third of development drop by an average 1.1°C, as seen in this study, sex ratios change from approximately >99% female to approximately 88% female for a nest incubating at 30.8°C in the absence of a cooling event. Increased cooling (e.g., 2°C) has an even stronger impact on sex ratios, particularly at temperatures that produce both males and females (Figure 1d). Recent and exciting new developments have shown that hatching sex can be determined directly from blood samples (Tezak et al., 2020), which may allow rigorous quantification of the shift in sex ratio caused by rainfall in the future. However, this positive effect of rainfall will not be sufficient if incubation temperatures continue to rise well above the pivotal temperature due to future climate warming. In addition, rainfall can have other impacts on incubating clutches beyond lowering sand temperatures. For example, increased precipitation may lead to increased mortality in clutches if incubation temperatures fall outside the optimal thermal range for embryonic development (Matsuzawa et al., 2002) or if nests are flooded.

Our work reiterates the concerns of Jensen et al. (2018) for the feminization of the Raine Island green turtle rookery, but also offers some hope for the future by showing the importance of isolated extreme rainfall events to produce male hatchlings. If extreme rainfall events occur at increased frequency in the future—as is projected for this part of Australia (CSIRO & BOM, 2018)—this may result in males still being produced on Raine Island despite warming temperatures. Further, increased incubation moisture resulted in improved locomotor abilities in freshwater turtle hatchlings (Miller, 1993; Miller et al., 1987) and the same may be true of sea turtles, given their reproductive and developmental similarities. Such an effect in sea turtles might result in a male dispersal phenotype with reduced risk of predation, at least when passing through predator‐dense nearshore waters (Gyuris, 2000). Crucially, the operational sex ratio (OSR, that is, the ratio of adult females to adult males on the breeding grounds) needed to maintain a viable population remains unknown. For example, once the OSR is >99% female, at what point (e.g., an OSR of 99.1%, 99.5%, or 99.9%?) will there be insufficient males to fertilize all the potential clutches and hence reduced hatchling production and population declines? Assessing clutch fertility with respect to the OSR is one approach that may allow this important question to be addressed.

With many studies reporting female‐biased sex ratios at rookeries across the world (Hays et al., 2014) and projections that these biases will be exacerbated (Hawkes et al., 2007; Jensen et al., 2018; Santidrián Tomillo et al., 2015), the prospect that extreme rainfall events can produce unexpected and large cohorts of males in the future is a welcome finding. Indeed, increased production of a few males can go a long way with sea turtles, as males breed multiple times and with multiple females during a breeding season (Lee et al., 2018). Male turtles also breed more often than females, who have longer interbreeding cycles (Hays et al., 2014). So, future irregular and large influxes of males into the breeding population, originating from extreme weather events during incubation, may help sustain a population for many decades after the extreme weather event occurred. This key conclusion is likely robust, especially given that sea turtles are long‐lived organisms and may reproduce multiple times during their life.

Given that Raine Island is thought to be producing >99% females, a single wet year when more males are produced may be important for helping to sustain the population for many years. We estimated that the extreme rainfall event that occurred in March 2019 affected <2% of all nests produced over the entire breeding season, yet as a result we estimate that between 5,600 and 10,400 additional males were produced. Therefore, considering the impact of extreme rainfall events on incubation temperatures may be essential when modeling how primary sex ratios are likely to change in the future.

Our results underline the importance of considering extreme rainfall events alongside more general warming when assessing the impacts of climate change on sea turtle populations (Santidrián Tomillo et al., 2020). While the amount of cooling experienced may vary between sites, the overarching conclusion that extreme events can cool nests and affect sex ratios is likely to hold for many sites around the world (Figure 1d). Ultimately, rainfall impacts need to be considered for hatchling survival and hatchling sex ratios. Only by doing so can we provide a holistic picture of likely climate change impacts on sea turtles. Our results also highlight the potential value of artificially cooling the sand by watering during a single point in the breeding season, that is, recreating the “perfect storm” if it does not occur naturally, as a way to ensure male hatchling production in the face of climate warming. However, we caution that if such management measures were to be put in place, careful considerations are needed to ensure the nests are not flooded and hatchling successes are not adversely impacted.

## CONFLICT OF INTEREST

The authors declare no conflicts of interest.

## AUTHOR CONTRIBUTIONS

**Jacques‐Olivier Laloë:** Conceptualization (equal); Formal analysis (lead); Investigation (equal); Methodology (equal); Writing‐original draft (lead); Writing‐review & editing (lead). **Jamie N. Tedeschi:** Conceptualization (equal); Investigation (equal); Methodology (equal); Writing‐review & editing (equal). **David T. Booth:** Conceptualization (equal); Investigation (equal); Methodology (equal); Writing‐review & editing (equal). **Ian Bell:** Conceptualization (equal); Investigation (equal); Methodology (equal); Writing‐review & editing (equal). **Andrew Dunstan:** Conceptualization (equal); Investigation (equal); Methodology (equal); Writing‐review & editing (equal). **Richard D. Reina:** Conceptualization (equal); Investigation (equal); Methodology (equal); Writing‐review & editing (equal). **Graeme C. Hays:** Conceptualization (equal); Formal analysis (equal); Investigation (equal); Methodology (equal); Writing‐original draft (equal); Writing‐review & editing (equal).

## Funding information

Data collection was funded by The Raine Island Recovery Project, a collaboration between BHP, the Queensland Government, the Great Barrier Reef Marine Park Authority, Wuthathi and Kemerkemer Meriam Nation (Ugar, Mer, Erub) Traditional Owners, and the Great Barrier Reef Foundation.

## Data Availability

Data that support the findings of this study are available from Dryad at https://doi.org/10.5061/dryad.0vt4b8gx9.
